# Developing a Bespoke Training Programme for Staff in a Psychiatric Intensive Care Unit: The Roxeth Ward Experience

**DOI:** 10.1192/bjo.2025.10274

**Published:** 2025-06-20

**Authors:** Mehtab Ghazi Rahman, Aquino Lieu, Alexandra Constantinescu

**Affiliations:** Cygnet Hospital Harrow, London, United Kingdom

## Abstract

**Aims:** Psychiatric Intensive Care Units (PICUs) present a unique and challenging environment for staff, requiring specialised knowledge and skills to manage complex clinical presentations and ensure patient safety. Currently, there is a gap in readily available, comprehensive training programmes specifically tailored for PICU staff. This project aimed to develop and evaluate a bespoke training programme for staff at Roxeth Ward PICU, addressing this gap and focusing on key clinical and operational challenges pertinent to the PICU setting. The programme sought to enhance staff competency, improve patient care, and create a more positive and therapeutic ward environment.

**Methods:** A bespoke training programme was designed and implemented for all staff at Roxeth Ward PICU, including nurses, psychiatrists, allied health professionals, and support staff. The programme incorporated a variety of interactive learning modalities to maximize engagement and knowledge retention. These included didactic lectures providing foundational knowledge, simulated scenarios (covering both mental health crises, such as managing acutely agitated patients, and medical emergencies, such as NMS), interactive group discussions to facilitate shared learning and problem-solving, and problem-based learning activities focused on real-world case studies encountered in the PICU. Topics covered a range of pertinent PICU issues, including the ward’s structure and processes, the function and purpose of operational/governance meetings, the safe and effective use of rapid tranquillisation and Acuphase, management of psychiatric emergencies, a comprehensive overview of the Mental Health Act and its legal implications for PICU practice, substance misuse management training, de-escalation techniques and strategies, quality improvement initiatives, the use of sensory modulation to create a therapeutic environment, understanding and applying knowledge of common mental health diagnoses, and the management of violence and aggression. Pre- and post-training assessments were conducted to evaluate the impact of the programme on staff knowledge, skills, and confidence.

**Results:** Quantitative feedback from the 35 participants demonstrated a substantial 64% improvement overall in knowledge and confidence following the training programme. These improvements were observed across all domains and topics covered in the training programme and a detailed breakdown of the results for each topic is included in the poster. Qualitative feedback from participants was overwhelmingly positive, with many staff highlighting the value of the interactive learning methods, particularly the simulated scenarios, in enhancing their understanding of complex clinical situations and improving their ability to apply theoretical knowledge to real-world practice. Participants specifically mentioned the increased confidence they gained in managing acutely agitated patients, understanding the legal implications of the Mental Health Act, and applying de-escalation techniques. The programme’s practical application to the PICU setting was consistently highlighted by participants, reinforcing the need for bespoke training tailored to this specialised area.

**Conclusion:** Our bespoke training programme represents a significant step towards addressing the gap in PICU-specific staff training. The diverse and interactive learning modalities, combined with the focus on key clinical and operational challenges specific to the PICU environment, appear to have a positive impact on staff knowledge, skills, and confidence. Further evaluation will explore the longer-term impact of the programme on staff practices, patient outcomes, and the overall ward environment. This model could serve as a valuable framework for other PICUs seeking to develop and implement tailored staff training programmes and contribute to best practice in mental health care.

